# The Bisphenols Found in the Ejaculate of Men Does Not Pass through the Testes

**DOI:** 10.3390/toxics10060311

**Published:** 2022-06-09

**Authors:** Michal Ješeta, Kateřina Franzová, Simona Machynová, Jiří Kalina, Jiří Kohoutek, Lenka Mekiňová, Igor Crha, Bartosz Kempisty, Marek Kašík, Jana Žáková, Pavel Ventruba, Jana Navrátilová

**Affiliations:** 1 Center of Assisted Reproduction, Department of Gynecology and Obstetrics, Masaryk University Brno and University Hospital Brno, 62500 Brno, Czech Republic; franzova.katerina@fnbrno.cz (K.F.); mekinova.lenka@fnbrno.cz (L.M.); crha.igor@fnbrno.cz (I.C.); zakova.jana@fnbrno.cz (J.Ž.); ventruba.pavel@fnbrno.cz (P.V.); 2 Department of Veterinary Sciences, Czech University of Life Sciences in Prague, 16500 Prague, Czech Republic; 3 Department of Urology, Faculty of Medicine, Masaryk University Brno and University Hospital Brno, 62500 Brno, Czech Republic; machynova.simona@fnbrno.cz (S.M.); kasik.marek@fnbrno.cz (M.K.); 4 RECETOX Centre, Faculty of Science, Masaryk University, 62500 Brno, Czech Republic; jiri.kohoutek@recetox.muni.cz (J.K.); jiri.kalina@recetox.muni.cz (J.K.); jana.navratilova@recetox.muni.cz (J.N.); 5 Department of Health Sciences, Faculty of Medicine, Masaryk University, 62500 Brno, Czech Republic; 6 Department of Veterinary Surgery, Institute of Veterinary Medicine, Nicolaus Copernicus University, 87-100 Torun, Poland; kempistybartosz@gmail.com; 7 Department of Histology and Embryology, Poznan University of Medical Sciences, 61-701 Poznan, Poland; 8 Department of Anatomy, Poznan University of Medical Sciences, 61-701 Poznan, Poland; 9 Prestage Department of Poultry Sciences, North Carolina State University, Raleigh, NC 27695, USA

**Keywords:** bisphenol A, bisphenol S, bisphenol F, endocrine disruptors, human, spermatozoa, IVF, vasectomy, biotransformation

## Abstract

Exposure to bisphenols is related to negative effects on male reproduction. The bisphenols exposure is associated with several modes of action including negative impact on the blood–testis barrier (BTB) in testes or direct effect on spermatozoa. Bisphenols have been detected in human seminal plasma, but the possible mechanism of seminal transfer of bisphenols is not clear. Some authors consider the transfer through the blood–testis barrier to be crucial. Therefore, in this work, we compared normozoospermic men and men after vasectomy who have interrupted vas deferens and their ejaculate does not contain testicular products. We measured the concentration of bisphenol A (BPA), bisphenol S (BPS) and bisphenol F (BPF) in the urine and seminal plasma of these men using liquid chromatography tandem mass spectrometry (LC/MSMS). We found that the ratio of urinary and seminal plasma content of bisphenols did not differ in normozoospermic men or men after vasectomy. From the obtained data, it can be concluded that the pathways of transport of bisphenols into seminal plasma are not primarily through the testicular tissue, but this pathway is applied similarly to other routes of transmission by a corresponding ejaculate volume ratio. To a much greater extent than through testicular tissue, bisphenols enter the seminal plasma mainly as part of the secretions of the accessory glands.

## 1. Introduction

Bisphenols are industrial chemicals widely used in consumer products (food packing materials, pipe linings, etc.) in order to increase their thickness and durability [1,2,3]. Because of their widespread consumer and commercial use, bisphenols are frequently detected in human samples including seminal plasma [4,5]. Their potential negative impact on male infertility [6], human oocyte development [7] or differentiation of foetal testicular tissue [8] have been described in several studies.

The most common pathway of their entry into organism is with food and drinks [9] that were stored in containers or bottles made of plastics containing for example bisphenol A (BPA) in its polymerized form. Mainly after exposure to high temperatures, its monomers can be released into the content and can be ingested into the body [10].

Regarding development of spermatozoa, there are several theories of negative effect of bisphenols on spermatogenesis. It is partially caused by binding to estrogen and androgen receptors in testicles [2] and partially by direct effect on spermatozoa and germinal cells [11]. Its effect on germinal epithelium through production of oxygen species and subsequent oxidative stress also is not irrelevant.

Despite rather a lot being known about potential effects of these substances on spermatogenesis, the way how bisphenols get into seminal plasma, where they directly affect sperm cells, is still not clear. Bisphenols were detected several times in blood, urine or seminal plasma [5]. Its content in seminal plasma is the most important in terms of spermatogenesis and is the main subject of this study.

Bisphenols that were not metabolized after entry to the organism, enter circulation in their unbound form and their lipophilic character enables their distribution to various tissues, such as adipose [12], brain [13], breast tissue [14], reproductive organs [15], prostate [16], maternal milk [17] or their transmembraneous transfer to foetal bodies [18]. From the tissues, they can be released to the systemic circulation again and be excreted with urine [15]. Besides urine, they can be detected in seminal plasma which is primarily a product of accessory sex glands. Therefore, it can be assumed that the source of bisphenols in seminal plasma is transport from blood circulation to accessory glands. However, it is not clear how transport through BTB in testicles contributes to the concentration of bisphenols in seminal plasma [19].

So far, most studies focused on the effect of bisphenols on spermatozoa evaluated bisphenols detected in urine. Results of the studies that detected bisphenols in urine and searched for correlations with deteriorated ejaculate quality, do not have consistent conclusions. One possible reason is that urine is not an optimal matrix for studies of this type. Metabolism of bisphenols is rather fast; most of the ingested bisphenols are excluded with urine within 24 h. However, urinary BPA concentrations do not directly inform about bioactive concentrations in specific tissues as mainly the conjugated forms, which are biologically inactive, are excluded with urine, while the unbound forms, that show high biological activity were detected in the seminal plasma.

For the evaluation of the effect of these substances on spermiogenesis, it is suitable to measure their concentration in seminal plasma directly since the concentration of bisphenols differs among blood, urine and seminal plasma. In addition, a negative correlation between concentration of BPA and sperm concentration, sperm count and morphology were observed only in samples of seminal plasma, not in blood plasma [5].

Although the transport of these substances to the ejaculate has not been studied thoroughly, their transport through the BTB in testicles has been mentioned frequently [19]. In men with vasectomy—artificial sterilization via surgical cross-section of vas deferens—products of testicular tissue are not present in the ejaculate. It lacks for example proteins specific for testicular tissue, such as testes specific protein 101 [20] or ACRV1 [21]. For this reason, these patients were selected as control for bisphenol biotransformation in organism and verification of hypothesis that bisphenols are not transferred to ejaculate from testicular tissue.

In this study, we have decided to verify the content of selected bisphenols in seminal plasma of non-operated men and men after surgical vasectomy. Subsequently, we performed comparison with concentration of these compounds in urine.

The aim of the work was to evaluate the importance of the transport of bisphenols through testicular tissue, since there are no products of testes in the ejaculate of men after vasectomy, including the products that pass the BTB.

## 2. Materials and Methods

### 2.1. Study Design

This prospective study included a total of 8 patients with normozoospermia and 8 men after surgical vasectomy with azoospermia at average age 32.4 years (from 22 to 41). The criteria for normozoospermia and azoospermia were defined according to the WHO manual (2010) [22]. Samples were collected between January 2020 and December 2021. The project was approved by the Ethics Committee of University Hospital Brno (Approval No. 10-170221/EK). All the patients agreed with their participation in the study and signed an informed consent. Semen sample was collected immediately after spermiogram analyses. The concentration of bisphenol A (BPA), bisphenol S (BPS) and bisphenol F (BPF) in the urine and seminal plasma of these men was detected by using liquid chromatography tandem mass spectrometry (LC/MSMS).

### 2.2. Semen and Urine Collection

Patients first time urinate to glass tube and after that semen samples were collected by masturbation after 3–8 days of sexual abstinence. Patients were asked to wash hands before masturbation. All samples were incubated at room temperature for 46–60 min until total liquefaction. Semen analyses was carried out in liquefied samples according to WHO guidelines [22]. Immediately after the spermiogram analyses, samples were centrifugated and seminal plasma was frozen in glass tube (−20 °C).

### 2.3. Vasectomy

All patients in the vasectomy group underwent classic open-ended vasectomy. The procedure is performed under general anaesthesia by an incision on each side of the scrotum. The cut is a maximum of 2 cm long. Following the preparation of the individual layers, the vas deferens is exposed and interrupted. A small portion of the vas deferens is removed. The ends of the vas deferens are ligated with a non-absorbable suture. The lumen is closed by coagulation. Finally, fascial interposition of the stumps is performed, i.e., the ends are immersed to different depths to increase the effect.

### 2.4. Bisphenol Detection in Urine

For the determination of total BPA, BPS and BPF, the urine samples were processed according to the methods described by Ye et al. [23,24]. Briefly, 500 μL of urine sample were mixed with 10 μL of 2 μg/mL internal standards solution, 20 μL of a β-glucuronidase/sulfatase solution (Helix pomatia, H1, 10,000 units/mL) and 100 μL of 1 M ammonium acetate buffer (pH 6.5). Samples were subsequently incubated for 1 h at 37 °C and sonicated. Thereafter, samples were extracted using solid phase extraction with Oasis WAX cartridges (Waters, Milford, MA, USA) that were previously washed and conditioned with water. Elution of bisphenols was carried out using methanol. The elution solvent was evaporated to dryness under a gentle stream of nitrogen gas. Prior to the analysis the residue was redissolved in 50% methanol (*v*/*v*). LC separation wad performed with an Agilent 1290 instrument (Agilent Technologies, Waldbronn, Germany) using Waters ™ ACQUITY ™ UPLC ™BEH C18 (100 × 2.1 mm, 1.7 µm) column and gradient elution with a mixture of 0.1 mM ammonium fluoride (A) and methanol (B) as mobile phases. The gradient profile was 50% B (0–1 min), then was linearly increased to 90% B (1–5 min), maintained at 90% B (5–7 min) and dropped to 50% B (7.01–10 min). The flow rate was 0.25 mL/min, and the injection volume was 5 μL. The elution time was 10 min. The column compartment was maintained at 30 °C. Bisphenols were measured with an Agilent 6495 Triple Quadrupole mass spectrometer (Agilent Technologies, Santa Clara, CA, USA) operating in the ESI-negative mode. Two MRM transitions were used for quantitative LC/MSMS analyses.

### 2.5. Bisphenol Detection in Seminal Plasma

For the determination of free BPA, BPS and BPF from seminal fluid, a mixture of toluene and ethyl acetate (50/50; *v*/*v*) was used. In general, 0.5 mL of seminal plasma was placed into a 5 mL Eppendorf tube and spiked with the mixture of isotopically labelled standards at level of 1 ng/mL. Then, 1 mL of cold acetonitrile was added for deproteination followed by vortexing and then sonicating for 10 min. Afterwards, the mixture was centrifuged at 2880× *g* for 10 min at 4 °C and the supernatant was removed and further evaporated to dryness under the gentle stream of nitrogen at 50 °C. The residue was reconstituted in a deionized water for subsequent extraction of bisphenols. For toluene/ethyl acetate extraction, 0.5 mL of toluene/ethyl acetate mixture (1 (thin space (1/6-em]]–(thin space (1/6-em))1, *v*/*v*) was added to 0.5 mL of redissolved residue in deionized water and shaken for 10 min. After the phase separation, 300 μL of the organic fraction was quantitatively transferred to HPLC glass vial and evaporated to dryness under gentle stream of nitrogen at 50 °C. The residue was redissolved in 50% methanol for bisphenol analysis. Chromatographic separation was performed on Waters ™ ACQUITY ™ UPLC ™ BEH C18 (100 × 2.1 mm, 1.7 µm) column using gradient elution with a mixture of 0.1 mM ammonium fluoride and methanol as mobile phases. Samples were analysed on Agilent 6495 Triple Quadrupole (Agilent Technologies, Santa Clara, CA, USA) operating in the ESI-negative mode. Two MS/MS transitions were used for quantitative LC-MS/MS analyses. Because it was impossible to obtain matrix that is blank for ever-present contaminants, non-spiked matrix was analysed simultaneously, and the measured concentration was subtracted from the concentration calculated in the samples.

## 3. Results

All the sample sets were analysed and concentrations of bisphenol A, bisphenol S and bisphenol F were determined in urine and seminal plasma. The results of bisphenol content in urine were standardized by conversion based on specific weight of urine according to [25]. Results of bisphenol content ratio in urine and seminal plasma are presented in Table 1. The levels detected in urine range from 0.24 to 5.15 ng/mL in case of BPA (Table 2), 0.03 to 6.7 ng/mL for BPS (Table 3) and 0.8–1.94 ng/mL for BPF (Table 4). The levels of bisphenols detected in seminal plasma are at similar level in the control patients and in the patients after vasectomy, ranging from 0.01 to 0.75 for BPA and from 0.01 to 0.85 for BPS. The detection frequency for BPF was low (18%, see Table 2) and these samples were removed from the further investigation. One value of BPS in urine below the limit of quantification (LOQ = 0.017 ng/mL) was replaced by LOQ/√2.

Since both the urine and seminal fluid concentrations did not differ from log-normal statistical distribution (Shapiro–Wilk’s test, *p*-value > 0.050), the ratio of urine–seminal plasma was calculated and log-transformed to obtain a normal distribution (again tested with Shapiro–Wilk’s test with *p*-value > 0.050). Mean of that ratio in the control group was 7.64 for BPA and 6.11 for BPS, mean in the patient with vasectomy was 5.64 for BPA and 3.39 for BPS. Finally, the differences in ratios between the control group and patients with vasectomy were tested using Welch’s t-test with *p*-values of 0.268 for BPA and 0.083 for BPS. These data were verified using Mann–Whitney’s test with *p* = 0.382 for BPA and *p* = 0.121 for BPS (see Table 1). None of the methods found any significant difference in ratios of urinary to seminal fluid bisphenols concentrations between the control group and patients with vasectomy.

## 4. Discussion

Bisphenols are ubiquitous substances, common in our environment. Their presence can affect a variety of physiological processes and they are often considered gametogenesis disruptors. Their effect is either direct on spermatozoa [11] or on germinal epithelium [26] or they can disrupt the BTB [27]. Until recently, many authors considered crossing the BTB to be the way for bisphenols to enter the ejaculate [19]. After ingestion in the organism, most BPA is metabolized by glucuronidation or sulfonation [28]. The greatest amount of BPA is metabolized in the liver, by UDP-glucuronosyltransferase 2B15 to inactive form BPA-glucuronide [29]. This form is water-soluble and is excreted in urine with a 5.4–6.4 h elimination half-life [30].

Unlike in plastic containers and plastic films in cans, where bisphenols are in polymerized forms, thermal paper contains up to milligrams of free BPA per gram [31,32]. Transdermal entry to the organism seems to be very important because it bypasses the metabolization of bisphenols in the liver. This route of transmission may significantly contribute to the total amount of bisphenols detected in urine or seminal plasma.

In addition, this transfer is accelerated by frequent use of hand disinfectants that can disrupt dermal barrier [31]. Because transdermal transfer bypasses the metabolization of bisphenols in the liver, this route of transmission may be significant for the total amount of bisphenols detected in urine or seminal plasma.

**Figure 1:**
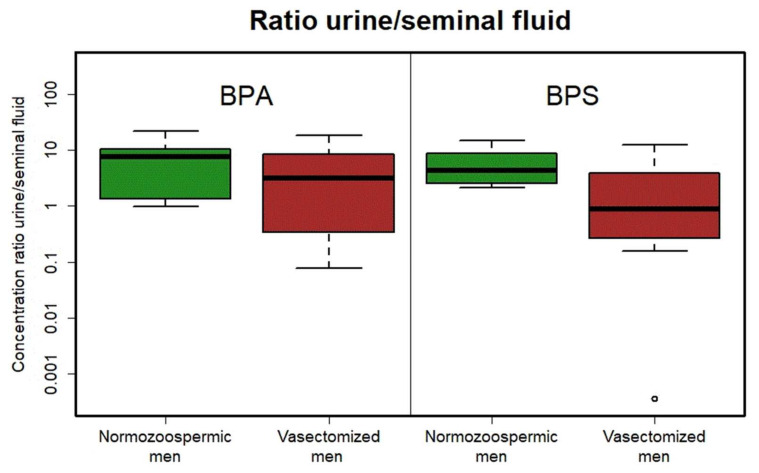
Boxplots of ratios between urinary and seminal plasma BPA (**left**) and BPS (**right**) concentrations in normozoospermic (green) and vasectomized (red) patients. ° indicates patient outside the main groupe.

Several authors have mentioned bisphenol transport primary via the blood–testis barrier in the testes [33]. In our study we did not find any differences in bisphenol urinal/seminal ratio in normozoospermic men and men after vasectomy (which ejaculate is without testicular products). This study, focused on detection of bisphenols in urine and seminal plasma, clearly demonstrated that the pathway for bisphenols to seminal plasma is not primarily via testicular tissue and that these substances are much more transported together with secrets of accessory glands (prostate, seminal vesicles and bulbourethral glands) that produce more than 95% of the human ejaculate volume. Our results suggest even the opposite trend, i.e., higher levels of bisphenols in seminal plasma than in urine in men after vasectomy in comparison with the control group. This is probably caused by a small set of patients with high individual differences. Indeed, in urine, total BPA was detected (i.e., also after metabolization), while only free BPA was detected in seminal plasma. Detection of multiple extreme values of the ratio of urinal/seminal bisphenols is observed in the group after vasectomy more often than in normozoospermic patients (Figure 1). This is probably due to the large dispersion of values and partly the role of chance. In this vasectomized men, we would expect there to be less bisphenols in ejaculate than urine and the urinal/seminal ratio would increase. However, we observe extreme values only in the opposite direction, i.e., higher concentrations of bisphenols in seminal plasma compared to urine. The final proportion of bisphenols contents in urine and seminal plasma can be affected by time of exposition or entrance of bisphenols to the organism. As mentioned before, there are two different biotransformation ways after per oral or transdermal exposition.

Seminal plasma is a mixture of secrets produced primarily by accessory glands, with 65–75% of volume produced by seminal vesicles, 20–30% by prostate and epididymis, 1% by bulbourethral and periurethral glands and 2–5% by testes [34]. Since the ratio of concentrations detected in seminal plasma and urine did not differ in normozoospermic men and men after vasectomy, it is highly probable that the transport of bisphenols to seminal plasma is mediated by accessory glands that contribute to the final composition of the ejaculate. This statement is supported by the finding that bisphenol F was detected only in three samples, but independently on the sample type and vasectomy performed. It is evident, that if a higher dose of bisphenols enters the organism, they reach the ejaculate no matter if there is a functional connection with testicular tissue or not.

Our results even did not show a significant decrease of bisphenol concentrations in vasectomized men compared to normozoospermic men. It suggests that transfer via testicular tissue is rather minor and that these substances enter seminal plasma via other transport pathways, from seminal vesicles, prostate or bulbourethral glands.

## 5. Conclusions

The importance of bisphenol transport from testicles via the BTB is negligible and its contribution to overall bisphenol content in seminal plasma is minimal. In conclusion, the transport of bisphenols to seminal plasma is mediated primarily via the accessory glands. The transport via testes is rather minor and its share on the total amount of bisphenols in seminal plasma is only small and corresponds to the share of seminal plasma produced in testicles.

## Figures and Tables

**Table 1:** Relative ratio of bisphenol content in urine and seminal plasma in men with normozoospermia and in men after vasectomy.

	Normozoospermic Men	Vasectomized Men
**Ratio of urinary BPA/seminal plasma BPA**	7.64	5.64
**Ratio of urinary BPS/seminal plasma BPS**	6.11	2.95
**Ratio of urinary bisphenols /seminal plasma bisphenols**	7.21	4.5

**Table 2:** Detection of bisphenol S (BPS) in all the observed patients in urine and in seminal plasma.

		Urine (ng/mL)	Seminal Plasma (ng/mL)
Normozoospermic men	patient 1	0.037	0.038
	patient 2	0.101	0.074
	patient 3	0.997	0.140
	patient 4	0.012	0.011
	patient 5	1.190	0.110
	patient 6	0.099	0.010
	patient 7	0.512	0.064
	patient 8	1.786	0.083
Vasectomized men	patient 1	5.105	0.280
	patient 2	0.418	0.089
	patient 3	1.010	0.204
	patient 4	0.039	0.507
	patient 5	0.530	0.749
	patient 6	0.268	0.132
	patient 7	3.230	0.226
	patient 8	0.120	0.749

**Table 3:** Detection of bisphenol A (BPA) in all the observed patients in urine and in seminal plasma.

		Urine (ng/mL)	Seminal Plasma (ng/mL)
Normozoospermic men	patient 1	0.085	0.017
	patient 2	0.247	0.041
	patient 3	0.030	0.013
	patient 4	0.134	0.009
	patient 5	0.078	0.006
	patient 6	0.022	0.008
	patient 7	0.077	0.037
	patient 8	0.335	0.090
Vasectomized men	patient 1	0.031	0.007
	patient 2	2.503	0.202
	patient 3	0.202	0.060
	patient 4	0.369	0.850
	patient 5	0.079	0.500
	patient 6	<0.017	33.683
	patient 7	0.430	0.329
	patient 8	0.302	0.516

**Table 4:** Detection of bisphenol F (BPF) in all the observed patients in urine and in seminal plasma. LOD = limit of detection.

		Urine (ng/mL)	Seminal Plasma (ng/mL)
Normozoospermic men	patient 1	<0.040	<LOD
	patient 2	<0.040	<LOD
	patient 3	<0.040	<LOD
	patient 4	<0.040	<LOD
	patient 5	<0.130	<LOD
	patient 6	<0.040	<LOD
	patient 7	<0.040	<LOD
	patient 8	0.810	<LOD
Vasectomized men	patient 1	<0.040	<LOD
	patient 2	1.94	<LOD
	patient 3	<0.040	<LOD
	patient 4	<0.040	<LOD
	patient 5	<0.040	<LOD
	patient 6	<0.040	0.991
	patient 7	<0.040	<LOD
	patient 8	<0.040	<LOD

## Data Availability

Not applicable.
